# Actual vs. perceived exertion during active virtual reality game exercise

**DOI:** 10.3389/fresc.2022.887740

**Published:** 2022-08-08

**Authors:** Trenton H. Stewart, Kirsten Villaneuva, Amanda Hahn, Julissa Ortiz-Delatorre, Chandler Wolf, Randy Nguyen, Nicole D. Bolter, Marialice Kern, James R. Bagley

**Affiliations:** ^1^Exercise Physiology Laboratory, Department of Kinesiology, San Francisco State University, San Francisco, CA, United States; ^2^Virtual Reality Institute of Health and Exercise, San Francisco, CA, United States

**Keywords:** video games, rating of perceived exertion, heart rate, exergames, energy expenditure, exercise intensity

## Abstract

**Background:**

Virtual exercise has become more common as emerging and converging technologies make active virtual reality games (AVRGs) a viable mode of exercise for health and fitness. Our lab has previously shown that AVRGs can elicit moderate to vigorous exercise intensities that meet recommended health benefit guidelines. Dissociative attentional focuses during AVRG gameplay have the potential to widen the gap between participants' perception of exertion and actual exertion.

**Objective:**

The aim of this study was to determine actual exertion (AEx) vs. perceived exertion (PEx) levels during AVRGs by measuring heart rate (HR) and ratings of perceived exertion (RPE) in two different settings.

**Materials and methods:**

HR and RPE were collected on participants (*N* = 32; age 22.6 ± 2.6) during 10 min of gameplay in LabS and GymS using the HTC VIVE with the following games played: Fruit Ninja VR (FNVR), Beat Saber (BS), and Holopoint (HP).

**Results:**

Participants exhibited significantly higher levels of AEx compared to reported PEx for all three AVRGs (Intensity): FNVR [AEx = 11.6 ± 1.8 (Light), PEx = 9.0 ± 2.0 (Very Light)], BS [AEx = 11.3 ± 1.7 (Light), PEx = 10.3 ± 2.1 (Very Light)], HP [AEx = 13.1 ± 2.3 (Somewhat Hard), PEx = 12.3 ± 2.4 (Light–Somewhat Hard)]. Additionally, participants playing in the GymS experienced significantly higher levels of AEx [12.4 ± 2.3 (Light–Somewhat Hard)] and PEx [10.8 ± 2.5 (Very Light–Light)] compared to the LabS [AEx = 11.6 ± 1.8 (Light), PEx = 10.3 ± 2.6 (Very Light–Light)].

**Conclusion:**

Perceptions of exertion may be lower than actual exertion during AVRG gameplay, and exertion levels can be influenced by the setting in which AVRGs are played. This may inform VR developers and health clinicians who aim to incorporate exercise/fitness regimens into upcoming ‘virtual worlds' currently being developed at large scales (i.e., the “metaverse”).

## Introduction

Only 20% of American adults meet the recommended levels of physical activity (PA) needed to improve health and reduce the risk of chronic diseases (1). Sedentary behavior in adulthood can be influenced by computer and television screen time, with >50% of adults playing video games on a regular basis (2). For example, time spent playing video games for the average college student has been reported to range from 10 to 35 hr/wk (3). Therefore, video games that require the user to be physically active during gameplay (sometimes termed “exergames”) are obvious targets for research due to video games' long standing as highly enjoyable and pleasurable leisure activities.

Exergaming, or the combination of exercise and video game play, has gained recent popularity for its potential to turn an otherwise traditional sedentary activity in video gaming into an active one. Innovative virtual reality (IVR) consoles place users in fully immersive 3D environments using head-mounted displays (HMDs) and motion tracking controllers (e.g., HTC VIVE, Oculus Quest). In this digital environment, players' activities are tracked in the virtual space and allow interactions between the player and other virtual objects beyond a physical boundary. This technology has led to new implementations of virtual reality games that require the user to be active during gameplay, termed 'active virtual reality games' (AVRGs). Recent work by our lab has shown significant increases in physiological variables such as heart rate (HR) and oxygen consumption (VO_2_) during AVRG play (4). Furthermore, AVRGs were found to elicit moderate-to-vigorous exercise intensities that meet the guidelines set forth by the American College of Sports Medicine (ACSM) (150 min/week of moderate or 75–150 min/week of vigorous aerobic exercise). The intensity of an exercise can be measured by measuring an individual's HR compared to their maximal HR (%HR_max_). Moderate intensity is between 65 and 75 % HRmax while vigorous intensity is between 76 and 96 % HRmax. Another way to examine exercise intensity is through Borg's ratings of perceived exertion (RPE) scale. The 15-point scale increases from 6 (no exertion at all) to 20 (maximal exertion) and has been used extensively to relate exercise workload and perception of effort (5–7). Furthermore, RPE corresponds to the HR range of a healthy young male (60-−200 beats/min) and HR can be predicted by multiply the RPE value by 10. Recently, Braun-Trocchio et al. (8) found that participants' HR and RPE both increased linearly with time and effort during a stepping task. This finding demonstrates that estimations of an individual's RPE can be reliably obtained using HR values.

According to Neumann and Moffitt (9), while exercising in a virtual environment, attention shifts away from internal stimuli (e.g., increased breathing, muscle pain) toward external sensory stimuli of the AVRG (e.g., game sounds, visual images). Therefore, exercise during AVRG may be affected by two categories of attentional focus. Dissociation represents an external attentional focus, mostly thought of as focusing on distractions from environmental stimuli, whereas association reflects an internal attentional focus, in which individuals concentrate on bodily sensations such as pain, breathing, or fatigue. Studies have found that when athletes adopt association strategies, compared to dissociation strategies during exercise, they report greater Ratings of Perceived Exertion (RPE) (10, 11). However, few studies have examined attentional focus of participants and RPE during AVRG play. One such study investigated attentional states during treadmill running in scenic vs. neutral induced virtual environments (9). Results showed that participants focused more on internal-associative cues while running in the neutral VR condition compared to the scenic VR condition. However, RPE did not differ between the scenic or neutral virtual environment groups over the trial. The authors suggested that the monoscopic virtual condition through the use of a projector did not yield a strong enough level of immersion to induce affective states that could influence the strength of psychological effects like RPE. Little data exist on how AVRG influences RPE, but one recent investigation found that RPE was lower during stationary cycling while playing AVRG vs. stationary cycling alone (12).

Although numerous studies have examined the effects of the virtual setting on cognitive strategies during exercise tasks, most have typically been conducted in a laboratory using traditional exercise apparatuses (e.g., cycle ergometers, treadmills) (13–15). Likewise, studies involving runners have shown different attentional focuses depending on the setting. Specifically, runners reported more dissociative attentive focus and lowered RPE in exercise settings that were considered more distracting (e.g., outdoor vs. indoor) (16–18). The interplay between exercise setting on RPE during AVRG participation has yet to be studied, but the effects of the virtual world could allow participants to partly dissociate from internal stimuli and focus on their digital surroundings (19). We wanted to ask if this dissociation would extend beyond the virtual boundary. The purpose of the study was to compare participants' actual exertion (AEx) to their perceived exertion (PEx) when playing 3 different AVRGs in different settings. We hypothesized that AEx would be higher than PEx due the enjoyable nature of AVRGs.

## Materials and methods

### Primary outcome

We observed differences between HR and RPE measurements during 10-min gameplay sessions across three VR games.

### Secondary outcome

We analyzed measurements of enjoyment between games and physical setting.

### Study design

The study was conducted using a within-subjects repeated measure design. The study protocols were approved by the Institutional Review Board at San Francisco State University (SFSU). Informed consent was obtained from all participants.

### Participants

Forty-seven college students between the ages of 18 and 39 were recruited. This age range was selected because the categorization of exercise intensity in this study is based on ACSM's guidelines for individuals up to 39 years of age. Participants were excluded from the study if they had a history of motion sickness or nausea related to virtual reality use, were using any drugs or medication that would affect metabolic processes of the body, or had contraindications to exercise as evaluated through a modified Physical Activity Readiness Questionnaire (PAR-Q). All participants were informed they could decline or withdraw from the study at any point and were assured of their confidentiality. Data analysis was performed only on data from participants who completed the entire study (32 total).

Participants completed a total of 3 visits in this study. Each visit was about 45 min in duration. During visit one, participants reported to the Exercise Physiology Lab (LabS) located on the SFSU campus where they were given a brief overview of the study procedures and asked to read and sign an Informed Consent Form. Afterwards, a modified PAR-Q was completed by participants along with a VR questionnaire that assessed experience playing video games and enjoyment of traditional forms of PA (e.g., cycling, weight-training). Height was recorded followed by weight and body composition measurements assessed using air displacement plethysmography (BOD POD: Life Measurement, Inc., Concord, CA). Participants then completed a self-paced graded exercise test (GXT) on a treadmill (T150, h/p/cosmos, Nussdorf-Traunstein, Germany) with a warm-up of 3 min at 0% grade, followed by increases of 2% grade every 2 min. Participants were only allowed to choose a speed of 5.0 mph or higher to ensure cardiorespiratory stress was achieved before muscular fatigue set in. Breath-by-breath measures of VO_2_ were recorded during the GXT using a metabolic gas analysis system (Quark CPET, CosMed Metabolic System; Life Measurement Inc.). Termination of the test was based on two of the three criteria being met: (1) VO2 or HR decreases with an increase in workload, (2) respiratory exchange ratio (RER) >1.14, and (3) RPE = 20. Once participants completed the self-paced graded exercise test, they underwent a familiarization session for the VR games.

### AVRG familiarization

**Figure 1:**
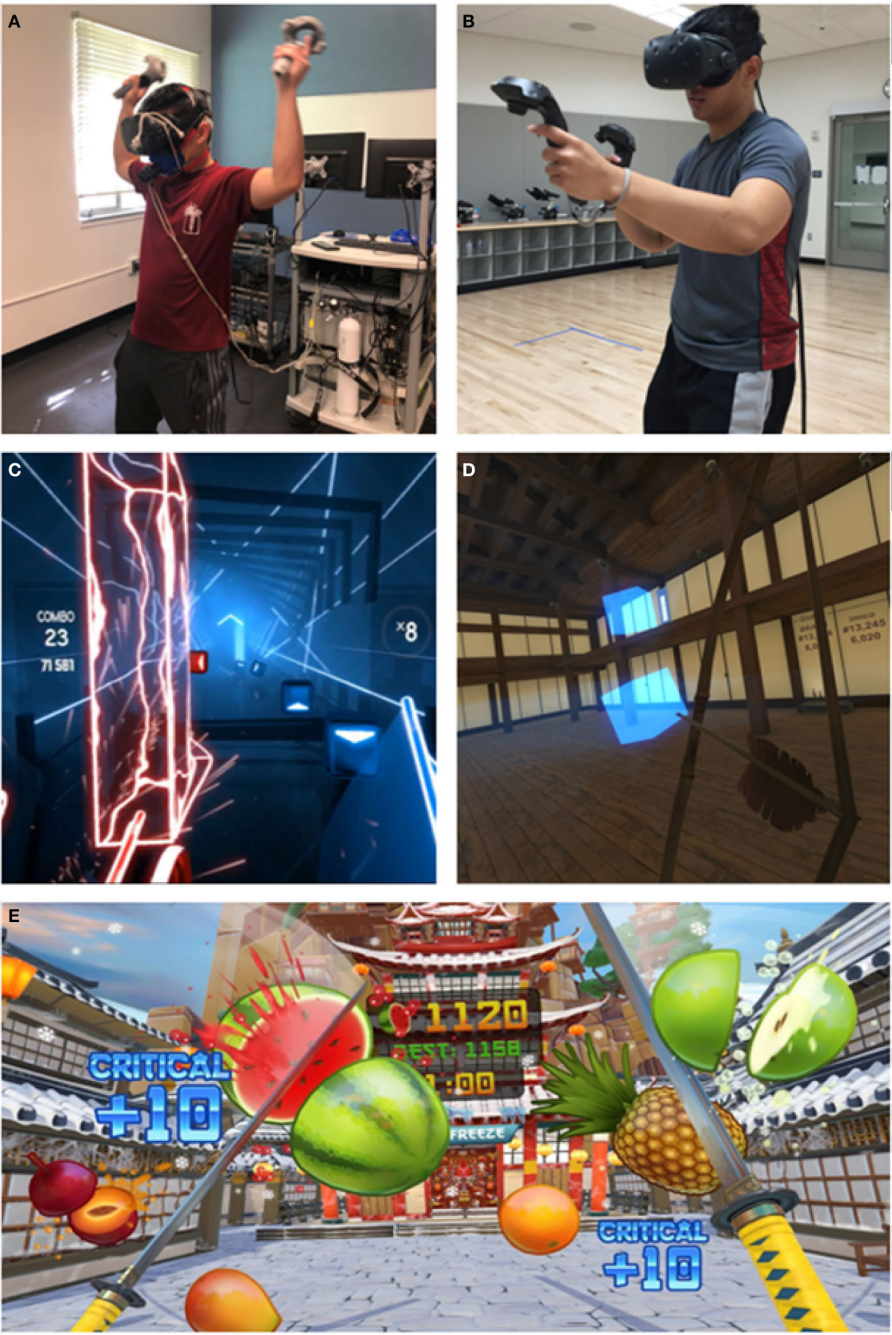
Experimental setup in exercise settings and in-game active virtual reality (VR) scenes. **(A)** Laboratory Setting (LabS), **(B)** Gym/Recreation Center Setting (GymS), **(C)** Gameplay during Beat Saber (Beat Games, Prague, CZ), **(D)** Gameplay during Holopoint (Azlan Studios, Florida, USA), **(E)** Gameplay during Fruit Ninja VR (Halfbrick Studios. Brisbane, AUS).

Participants underwent a familiarization session with the AVRGs while wearing the HTC VIVE equipment and a HR monitor as described previously (4). Trained research assistants optimized the fitting of all wearable equipment to ensure participant comfort during gameplay. Participants were given details on the button layout of the VIVE VR controllers after fitting. Player movement in the real world is tracked by built-in sensors located on the HMD and controllers and then translated into movement in the virtual environment via room-scale tracking technology. Two standing tripod-mounted base stations located in the room designated the boundaries and physical space participants could perform their movements in. All AVRGs were launched by the researchers from Steam, a digital video game platform. Before each AVRG was played, a synopsis was read out loud to participants that explained the purpose of the game and button/controller use during gameplay. Participants played a total of 3 AVRGs with 5 min sessions allotted for each game; games included Fruit Ninja VR (FNVR), Beat Saber (BS), and Holopoint (HP) (see Figure 1). These AVRGs were chosen because they have been shown to elicit different exercise intensities as described by the VR Institute of Health and Exercise. Specifically, FNVR is equivalent to exercising on an elliptical, BS and HP equivalent to playing tennis. The order of AVRGs played was randomized. Once the 5 min familiarization session concluded for each AVRG, participants were given sufficient time to rest in between games (i.e., HR reached 50% of max). Upon completion of the familiarization session, participants were scheduled to come back to the lab to complete their first randomized experimental session no <48 h later. Followed by another visit, no <48 h later, to complete the 2nd experimental session.

### Experimental protocol

Participants completed two randomized experimental sessions in different exercise settings. The exercise settings included the Exercise Physiology Laboratory (LabS) and the Mashouf Wellness Center (GymS) both located on the San Francisco State University campus (*Note:* GymS is a recreation center with a gym, group fitness rooms, pool, basketball courts, etc.). Each of the visits were separated by a minimum of 24 h. At the start of each session, participants were fitted with Polar H10 (Polar, Kemepele, FI) chest strap heart rate monitors that were synced to the Polar Beat application on a smartphone. HTC VIVE headset and controllers were equipped by participants and the wires connecting the headset to the computer were placed behind participants to avoid their frontal region and arms. Order of the AVRGs was randomized using an online randomizer tool. Participants played each AVRG for 10 min with 5 min of rest (chair-sitting) between games. The amount of time to rest was chosen to allow HR to return within 10% of resting level. During rest, participants completed 1–10 Likert scales for enjoyment and were asked to recall their highest perceived exertion (RPE) during the 10 min session. Participants' oxygen consumption (VO_2_) was measured only in the LabS using a facemask connected to a metabolic cart (CosMed Metabolic System; Life Measurement, Inc.).

### Heart rate: Single-lead electrocardiogram

A Polar H10 (Polar, Kemepele, FI) single lead electrocardiogram (ECG) worn by participants tracked heart rate during PA. The H10 monitors are small lightweight and waterproof single lead ECGs with a soft adjustable textile strap. The HR monitor was fastened around participants' chest and making skin contact. Single lead ECGs such as the H10 have been validated as a criterion measure for heart rate against other HR monitors such as a 12-lead ECG (20). Pilot data from our lab had shown that participants reached a steady state during AVRG gameplay around minute 4 into exercise. We expected participants to have reached steady state and their highest levels of perceived exertion from the 5th to 10th minute of gameplay. Consequently, HR was recorded as the average of the last 5 min of gameplay.

### Ratings of perceived exertion: The Borg scale of perceived exertion

At the end of each 10 min AVRG session, participants were asked to verbally provide a rating of their perceived exertion, using the 6–20 Borg Scale (see Table 1). Specifically, participants were asked to recall what their highest perceived effort was during the entire 10 min session and the number was recorded as participant's PEx. Retrospective RPE self-assessments have been shown to be valid forms of measurement against RPE self-assessments during exercise (22).

**Table 1:** Rating of Perceived Exertion (RPE) from 6-20 based on estimated heart rate [adapted from Borg (21)] (heart rate max, HRmax).

**BORG RPE**	**Intensity**	**HR_max_ (%)**
6	No exertion at all	20–39
7	Extremely light	
8		40–59
9	Very light	
10		
11	Light	60–69
12		
13	Somewhat hard	70–79
14		
15	Hard	80–89
16		
17	Very hard	90–99
18		
19	Extremely hard	100
20	Maximal exertion	

### Oxygen consumption (VO_2_)

Measurements of oxygen consumption are considered the gold-standard of cardiopulmonary performance assessments for exercise capacity and metabolic cost. A person's maximal oxygen consumption (VO_2max_) can be used to predict cardiovascular (CV) mortality and morbidity (23). Measurements of oxygen consumption are commonly measured in exercise labs to determine one's exercise intensity. However, VO_2_ measurements require expensive machinery and a 6ft sampling line connected to gas analyzers to conduct exercise tests. Therefore, measurements in field settings or gym settings are not commonly measured.

### Actual exertion (AEx)

The RPE scale is designed based on a linear relationship to the expected HR for a given exercise intensity (RPE = HR/10). A multitude of studies have examined this relationship utilizing the 6–20 scale as an indicator for perceived exertion depending on exercise intensity (24, 25), age (26, 27), gender (28, 29), and ethnicity (30). The results of these studies confirm the positive linear correlation between HR and RPE. Participants AEx was obtained by dividing their HR by 10 (AEx = HR/10).

### Data analysis

Data were analyzed using IBM SPSS Statistics 28. The means and standard deviations for each variable and condition were calculated and tested for normal distribution using the Shapiro-Wilk W-test. To answer our research questions, two 3 (games: FNVR, BS, HP) x 2 (settings: LabS, GymS) x 2 (measures: AEx, PEx) ANOVAs were used to determine any significant main effects or interactions on (a) exertion and (b) enjoyment. A *p*-value of 0.01 was used based on Bonferroni correction, and effect sizes were reported using partial eta squared.

## Results

### Demographics

Overall, 32 individuals participated in 3 visits, including a day of familiarization and experimental procedure in two settings. Baseline characteristics of the participants are presented in Table 2. Age and BMI were comparable between the two genders; however, males had, on average, reported more time spent playing video games compared to their female counterparts. A Chi-square test for independence (with Yates Continuity Correction) confirmed a significant association between gender and video game play, *X*^2^ (1, *n* = 32) = 10.8, *p* = 0.001, phi = 0.65.

**Table 2:** Participant characteristics.

**Characteristic**	**Male (*n* = 16)**	**Female (*n* = 16)**
Age, *y*	22.9 (2.8)	22.3 (2.3)
BMI, (kg/m^2^)	24.7 (4.2)	23.5 (3.8)
HR_rest_ bpm	68.4 (8.7)	72.8 (8.9)
VO_2max_ (mL/min/kg)	47.0 (5.6)	42.7 (5.0)
**Video game play**		
≥1 time per week	15 (94%)	5 (31%)
No video game play	1 (6%)	11 (69%)

Values other than Video game play are reported as mean (SD).

SD, standard deviation; y, years; BMI, body mass index; kg, kilogram; m, meter; bpm, beats per minute; VO_2max_, maximal oxygen consumption; mL, milliliters.

### Perceived and actual exertion

**Figure 2:**
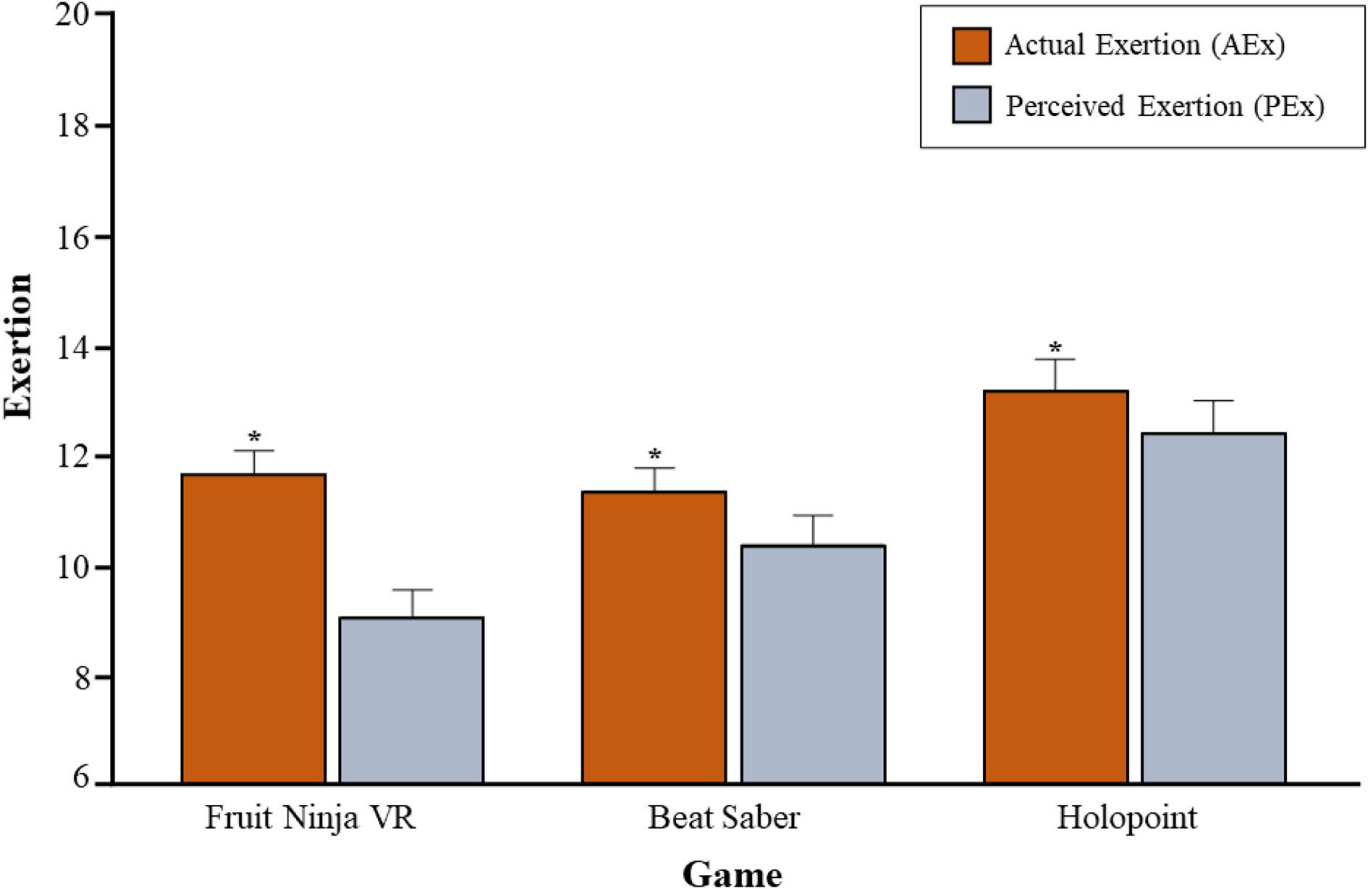
Actual exertion (AEx; mean ± SD) and perceived exertion (PEx; mean ± SD) by game. [Fruit Ninja VR (FNVR), Beat Saber (BS), and Holopoint (HP)]. AEx was obtained based on the positive linear relationship between HR and RPE (AEx = HR/10). *P < 0.05 vs. PEx of same game.

There were differences between AEx and PEx across all three gaming conditions (see Figure 2). A significant main effect was observed for game, Wilks Lambda = 0.31, *F*_(2,30)_ = 34.39, *p* = <0.001, partial eta squared = 0.7, with *post-hoc* testing indicating participants' exertion levels were highest playing HP, followed by BS, and then FN (see Table 3). Furthermore, there was a significant main effect for measure, Wilks Lambda = 0.6, *F*_(1,31)_ = 21.29, *p* = <0.001, partial eta squared = 0.41, with AEx levels higher than PEx across game or setting. Lastly, there was a significant interaction effect between game and measure, Wilks Lambda = 0.44, *F* (2,30) = 18.81, *p* = <0.001, partial eta squared = 0.56, with all three games having higher measures of AEx compared to PEx.

**Table 3:** Measures of physiological responses (heart rate, HR; estimated rating of perceived exertion, estimated RPE) and psychological responses (rating of perceived exertion, RPE; enjoyment) during active virtual reality gaming (AVRG) in different settings (Laboratory, LabS; Gym/Recreation Center, GymS).

		**HR**	**Estimated RPE**	**RPE**	**Enjoyment**
LabS	Fruit Ninja	113 ± 16.2	11.2 ± 1.5	8.7 ± 1.8	7.8 ± 1.7
	Beat Saber	109 ± 13.2	11.0 ± 1.3	10.3 ± 2.1	8.2 ± 1.7
	Holopoint	125 ± 21.2	12.7 ± 2.1	12.0 ± 2.5	8.8 ± 1.9
GymS	Fruit Ninja	119 ± 20.4	11.9 ± 2.0	9.3 ± 2.1	8.1 ± 1.4
	Beat Saber	116 ± 19.0	11.6 ± 1.9	10.4 ± 2.1	8.4 ± 1.4
	Holopoint	135 ± 24.1	13.6 ± 2.4	12.7 ± 2.2	9.0 ± 1.1

Data are means ± SD.

### Exercise setting

The setting where AVRGs were played had an effect on overall exertion levels, as indicated by a significant main effect for setting, Wilks Lambda = 0.8, *F*_(1,31)_ = 7.83, *p* = 0.009, partial eta squared = 0.2, with higher levels of exertion in the GymS compared to the LabS setting.

### Enjoyment

Only two small to moderate negative correlations were found between exertion and enjoyment: one between AEx and enjoyment rating of playing FNVR in LabS (*r* = −0.357, *p* = 0.45) and one between PEx and enjoyment rating of playing FNVR in LabS (*r* = −0.388, *p* = 0.028). All other correlations between enjoyment and either measure of exertion (AEx or PEx) did not reach statistical significance.

A 3 (games: FNVR, BS, HP) x 2 (settings: LabS, GymS) ANOVA with enjoyment as the dependent variable revealed a significant main effect for game, Wilks Lambda = 0.69, *F*_(2,30)_ = 6.83, *p* = 0.004, partial eta squared = 0.31. *Post-hoc* analyses indicated that participants enjoyed HP significantly more than BS or FN. However, there was no significant difference between BS or FN for enjoyment (*p* > 0.05). The main effect comparing settings was not significant, *F* (1, 31) = 1.74, *p* = 0.2, partial eta squared = 0.053, suggesting no differences in enjoyment between LabS and GymS. Furthermore, no significant interaction effect was found between game and setting for enjoyment, *F* (2, 30) = 0.051, *p* = 0.95, partial eta squared = 0.003.

## Discussion

In this study, we aimed to investigate the differences in actual exertion (AEx) measures *via* HR, and perceived exertion (PEx) measured *via* RPE, while playing AVRGs. This study further sought to compare exertion levels in a lab and gym to identify whether setting would affect participants' energy expenditure. The data showed higher AEx levels compared to PEx while playing AVRGs, as well as higher overall exertion levels in GymS compared to LabS. To our knowledge, this is the first study investigating AEx and PEx in different settings using an HMD VR device.

Only one study to date has examined the differences between actual and perceived exertion during AVRG gameplay. A study conducted by Yoo et al. (31), at the University of Sydney compared young adults' %HR_max_ and RPE during three AVRGs (FNVR, HP, Hot Squat). Participants played each game for a time range of 5–10 min with resting sessions lasting 2 to 10 min between games. Data from the study showed that RPE levels were matched correctly for FNVR but underestimated for HP based on %HRmax levels and their exercise intensity classifications (light intensity, moderate intensity, vigorous intensity). The findings from their study partially align with our study results where underestimations of exertion were found compared to actual exertion levels.

Previous work by our lab (4) observed similar results when comparing PEx against physiological variables. Participants reported lower RPE levels for 2 of the 3 games played compared to %VO_2_R and METs. In our current investigation, AEx was higher for all gaming conditions (FNVR, BS, HP) compared to PEx. A lower PEx compared to AEx has the potential to allow participants to engage in PA for longer durations or at higher intensities. In other words, if an individual perceives themselves as not exerting that much energy, there is a decreased likelihood that feelings of pain or fatigue may inhibit how long or how hard they are working out. Accordingly, boredom and fatigue have been shown to be key determinants in PA engagement and adherence (32, 33). While the general goal behind the implementation of VR technology (in an exercise context) is to increase an individual's involvement and adherence, research has shown that this exercise technology diverts attention away from unpleasant bodily sensations (34, 35), thus delaying the onset of boredom and fatigue. In this current study, attentional focus was not one of the measurements. However, we interpreted the underestimations of exertion to be reflective of dissociative attentional states stemming from the use of fully-immersive VR HMDs. Future research could assess more subjective measures of attentional states utilizing items like the *Measure of Attentional Focus* (MAF) (36), and *The Immersive Tendencies Questionnaire* (ITQ) (37). Alternatively, future research could examine attentional focus between an HMD and non-HMD VR-based exercise.

One of the main tenets of physiotherapy rehabilitation is having patients return to their normal level of activity prior to injury or disorder. However, conventional interventions in physiotherapy treatment consist of mundane, equipment-prohibitive activities (38). VR-based rehabilitation has the potential to overcome these challenges by allowing continual changes in task variables during treatment sessions (size, speed, environment, etc. (39). In this study, participants reported their perceptions of exertion to be less than their actual exertion during VR exercise. This, combined with the potentially motivating aspects of VR-based rehabilitation, can increase engagement, and may result in longer durations of exercise during cardiac rehabilitation programs. Studies examining this topic have found results that point toward increased engagement and motivation during rehabilitation. Although rehabilitation was not a goal in our study, the results were positive for physical exercise improvements similar to Garcia-Bravo et al. (40), who found increases in exercise intensity and resistance to fatigue during virtual reality supplemented cardiac rehabilitation.

This study examined AVRG exercise in two settings (LabS and GymS), uniquely contributing to the literature on this topic because no study to date has examined both physiological and psychological measures across these two settings. Our participants reported overall higher levels of exertion in the GymS compared to the LabS. However, enjoyment did not differ between settings, which partially supports our hypothesis that exertion and enjoyment would not differ between settings. One possible explanation for the former finding is the methodological approach we took to measure exertion in the LabS through VO_2_ requirements while playing AVRGs. Because VO_2_ could not be measured in the GymS, participants only wore chest-strap HR monitors and were not connected to a metabolic system. We believe that the absence of a tethered connection to a metabolic system allowed participants to move more freely in their play space. Therefore, in our current study we interpreted the higher levels of exertion in the GymS to be attributed to the freedom of movement participants were allowed compared to the LabS. Moreover, analyses comparing HR between settings confirmed higher HR levels in the GymS compared to LabS. This finding echoes the linear relationship between RPE and HR found in previous studies (41, 42). Thus, similar conclusions can be made about the connection between HR and RPE while playing AVRGs.

Enjoyment has been shown to be a significant determinant of PA behaviors, specifically, as enjoyment increases so does PA participation (43, 44). Numerous studies have found that AVRGs increase participants' enjoyment while exercising (34, 45, 46). However, these studies have typically employed 2D monoscopic virtual displays (i.e., Nintendo Wii, Dance Revolution), with a scarcity of studies utilizing new commercially available HMDs VR devices. This study used the HTC VIVE, which is a commercially available VR system equipped with HMDs and full-room body tracking. The results from this study showed relatively high scores for enjoyment for all three gaming conditions in both settings. Interestingly, the most intense AVRG (HP) was also perceived as the most enjoyable by participants in this study, which is inconsistent with previous research that has found enjoyment to be inversely related to exercise intensity (47, 48). However, a study involving a VR-based exercise experience found that as participants became more skilled in the exercise task, their enjoyment increased (49). In this study, increases in enjoyment for the higher-intense AVRG was interpreted to also be the result of participant skill level in game. In other words, if participants were unable to progress to higher levels in the game, they rated their enjoyment relatively low.

Our lab has shown VR-based exercise intensities to reach similar levels seen in conventional exercises (moderate-to-vigorous intensity). VR can be used to produce cardiorespiratory benefits that match typical rehabilitation efforts with few additional undue risks involved. However, virtual environments can induce symptoms of motion sickness including dizziness, nausea, and headache. In a study involving the latest head-mounted display VR systems, Koslucher et al. (50), had 22% of their participants report feelings of motion sickness after 15 min of use. Surprisingly, VR-induced motion sickness is typically preceded by patterns of postural sway in participants. Koslucher et al. (50) found that the incidence of motion sickness stemming from visual motion occurred at higher rates when participants had greater anterior-posterior axis sway velocity. Therefore, considerations for the side effects during VR-based activities can, for the most part, be reduced by screening for postural sway in participants before tasks.

Limitations to this study included the utilization of wired devices in data collection. The HTC VIVE headset requires a wired input to a desktop computer which runs the software and games. This 6-foot cable allowed limited freedom of movement in a boundary play space for participants. Additionally, a 6-foot sampling line was connected from a metabolic cart to a VO_2_ facemask that participants wore to collect VO_2_ data. Consequently, these may have limited participants' movements while playing AVRGs. For example, in HP, movement around the play space is required to dodge incoming missiles and make whole-body turning motions. Furthermore, some participants were visibly hesitant to make big body movements that may have caused the cable to become untethered to the desktop computer. To minimize both issues, researchers moved the metabolic cart along with participants' movements to allow as much slack as possible in the sampling line and VR cable. Already, standalone VR HMDs without cables are available to consumers that future research examining exercising in VR may benefit from using. This modification utilizing untethered HMDs would create a setting similar to how other exercise tasks are performed, increasing the external validity of both AEx and PEx assessments. Finally, we chose specific options from the list of in-game settings from each game in an attempt to design a 10 min gameplay session that best resembled what participants would play at home. Therefore, the generalizability of data from the game settings we chose to other game settings remains unknown.

## Conclusion

As VR technology becomes more advanced, new “virtual worlds” running perpetually alongside the real world are being developed at large scales (i.e., the “metaverse”) with upcoming societal impacts on work, play, health, and exercise. Research on AVRG may be used to inform developers implementing fitness regimens into these virtual worlds. Our data shows that AVRG can be used to meet physical activity guidelines for health (4), and that AVRGs may offer individuals a mode of exercise that elicits lower perceived exertion than would be observed during traditional exercise. Future research should continue to investigate the complex interplay between human physiology and psychology during exercise in VR.

## Data availability statement

The raw data supporting the conclusions of this article will be made available by the authors, without undue reservation.

## Ethics statement

The studies involving human participants were reviewed and approved by Institutional Review Board for San Francisco State University. The patients/participants provided their written informed consent to participate in this study. Written informed consent was obtained from the individual(s) for the publication of any potentially identifiable images or data included in this article.

## Author contributions

TS, JB, and MK contributed to the conception and design of the study. TS, KV, AH, JO-D, CW, and RN collected the data. TS and NB performed the statistical analysis. TS wrote the first draft of the manuscript, and it was reviewed and edited by JB, NB, and MK. All authors contributed to manuscript revision, read, and approved the submitted version.

## Funding

This study was funded by a Joint Venture Grant (California State University; CSUPERB) to JB and the Virtual Reality Institute of Health and Exercise.

## Conflict of interest

The authors declare that the research was conducted in the absence of any commercial or financial relationships that could be construed as a potential conflict of interest.

## Publisher's note

All claims expressed in this article are solely those of the authors and do not necessarily represent those of their affiliated organizations, or those of the publisher, the editors and the reviewers. Any product that may be evaluated in this article, or claim that may be made by its manufacturer, is not guaranteed or endorsed by the publisher.

## References

[1] American College of Sports Medicine (2017). ACSM's Guidelines for Exercise Testing and Prescription. 10th ed. Philadelphia, PA: Lipincott Williams and Wilkins.

[2] Wittek CT Finseras TR Pallesen S Mentzoni RA Hanss D Griffiths MD. Prevalence and predictors of video game addiction: a study based on a national representative sample of gamers. Int J Ment Health Addict. (2016) 14:672–86. 10.1007/s11469-015-9592-8 27688739 PMC5023737

[3] Wack E Tantleff-Dunn S. Relationships between electronic game play, obesity, and psychosocial functioning in young men. CyberPsychol Behav. (2009) 12:241–44. 10.1089/cpb.2008.0151 19006465

[4] Gomez DH Bagley JR Bolter N Kern M Lee CM. Metabolic cost and exercise intensity during active virtual reality gaming. Games Health J. (2018) 7:310–6. 10.1089/g4h.2018.0012 30325233

[5] Borg G. Perceived exertion as an indicator of somatic stress. Scand J Rehabil Med. (1970) 2:92–8. 5523831

[6] Tenenbaum G Connolly CT. Attention allocation under varied workload and effort perception in rowers. Psychol Sport Exerc. (2008) 9:704–17. 10.1016/j.psychsport.2007.09.002

[7] Hall EE Ekkekakis P Petruzzello SJ. Is the relationship of RPE to psychological factors intensity-dependent? Med Sci Sports Exerc. (2005) 37:1365–73. 10.1249/01.mss.0000174897.25739.3c 16118584

[8] Braun-Trocchio R Williams A Harrison K Warfield E Renteria J. The effects of heart rate monitoring on ratings of perceived exertion and attention allocation in individuals of varying fitness levels. Front Sports Act Living. (2022) 3:798941. 10.3389/fspor.2021.798941 35072065 PMC8777058

[9] Neumann DL Moffitt RL. Affective and attentional states when running in a virtual reality environment. Sports. (2018) 6:71. 10.3390/sports6030071 30060451 PMC6162466

[10] Russell WD Weeks DL. Attentional styles in ratings of perceived exertion during physical exercise. Percept Mot Skills. (1994) 78:779–83. 8084691 10.1177/003151259407800319

[11] Connolly C Janelle C. Attentional strategies in rowing: Performance, perceived exertion, and gender considerations. J Appl Sport Psychol. (2003) 15:195–212. 10.1080/10413200305387

[12] McDonough DJ Pope ZC Zeng N Liu W Gao Z. Comparison of college students' blood pressure, perceived exertion, and psychosocial outcomes during virtual reality, exergaming, and traditional exercise: An exploratory study. Games Health J. (2020) 9:290–6. 10.1089/g4h.2019.0196 32609004

[13] Annesi JJ Mazas J. Effects of virtual reality-enhanced exercise equipment on adherence and exercise-induced feeling states. Percept Mot Skills. (1997) 85:835–44. 9399288 10.2466/pms.1997.85.3.835

[14] Peng W Crouse JC Lin JH. Using active video games for physical activity promotion: a systematic review of the current state of research. Health Educ Behav. (2013) 40, 171–192. 10.1177/1090198112444956 22773597

[15] Gillman AS Bryan AD. Effects of performance **vs.** game-based mobile applications on response to exercise. Ann Behav Med. (2016) 50:157–62. 10.1007/s12160-015-9730-3 26362539

[16] Reich AH Queathem EJ. Setting, age, and intensity influence responses to exercise in young endurance runners. Percept Mot Skills. (2020) 127:533–54. 10.1177/0031512520903907 32036749

[17] Harte JL Eifert GH. The effects of running, environment, and attentional focus on athlete's catecholamine and cortisol levels and mood. Psychophysiology. (1995) 32:49–54. 7878169 10.1111/j.1469-8986.1995.tb03405.x

[18] Pennebaker JW Lightner JM. Competition of internal and external information in an exercise setting. J Pers Soc Psychol. (1980) 39:165–74. 10.1037/0022-3514.39.1.165 7411392

[19] Aardema F O'Connor K Cote S Taillon A. Virtual reality induces dissociation and lowers sense of presence in objective reality. Cyberpsychol Behav Soc Netw. (2010) 13:1–7. 10.1089/cyber.2009.0164 20712501

[20] Dooley EE Golaszweski NM Bartholomew JB. Estimating accuracy at exercise intensities: a comparative study of self-monitoring heart rate and physical activity wearable devices. JMIR Mhealth Uhealth. (2017) 5:e34. 10.2196/mhealth.7043 28302596 PMC5374271

[21] Borg G. Borg's Perceived Exertion and Pain Scales. Champaign, IL: Human Kinetics (1998).

[22] Milanez VF Spiguel-Lima MC Gobatto CA Perandini LA Nakamura FY Ribeiro LFP. Correlates of session-rate of perceived exertion (RPE) in a karate training session. Sci Sports. (2011) 26:38–43. 10.1016/j.scispo.2010.03.009

[23] Jones S Tillin T Williams S Coady E Chaturvedi N Huges AD. Assessment of exercise capacity and oxygen consumption using a 6 min stepper test in older adults. Front Physiol. (2017) 8:408. 10.3389/fphys.2017.00408 28659823 PMC5469915

[24] Zinoubi B Zbidi S Vandewalle H Chamari K Driss T. Relationships between rating of perceived exertion heart rate and blood lactate during continuous and alternated-intensity cycling exercises. Biol Sport. (2018) 35:29–37. 10.5114/biolsport.2018.70749 30237659 PMC6135975

[25] Cochrane KC Housh TJ Bergstrom HC Jenkins NDM Johnson G Schmidt RJ. Physiological responses during cycle ergometry at a constant perception of effort. Int J Sports Med. (2015) 36:466–73. 10.1055/s-0034-1396826 25700102

[26] Lupo C Tessitore A Gasperi L Gomez MAR. Session-RPE for quantifying the load of different youth basketball training sessions. Biol Sport. (2017) 34:11–7. 10.5114/biolsport.2017.63381 28416891 PMC5377554

[27] Morishita S Tsubaki A Nakamura M Nashimoto S Jack B Onishi H. Rating of perceived exertion on resistance training in elderly subjects. Expert Rev Cardiovasc Ther. (2019) 17:135–42. 10.1080/14779072.2019.1561278 30569775

[28] Haddad M Stylianides G Djaoui L Dellal A Chamari K. Session-RPE method for training load monitoring: Validity, ecological usefulness, and influencing factors. Front Neurosci. (2017) 11:612. 10.3389/fnins.2017.00612 29163016 PMC5673663

[29] Giancotti GF Fusco A Rodio A Capranica L Cortis C. Energy expenditure and perceived exertion during active video games in relation to player mode and gender. Kinesiology. (2018) 50:18–24. 10.26582/k.50.1.3

[30] Leung RW Leung ML Chung PK. Validity and reliability of cantonese-translated rating of perceived exertion scale among hong kong adults. Percep Motor Skills. (2004) 98:725–35. 10.2466/pms.98.2.725-735 15141939

[31] Yoo S Ackad C Heywood T Kay J. Evaluating the actual and perceived exertion provided by virtual reality games. In: Proceedings of the 2017 CHI Conference Extended Abstracts on Human Factors in Computing Systems (2017).

[32] Wolff W Bieleke M Martarelli CS Danckert J. A primer on the role of boredom in self-controlled sports and exercise behavior. Front Psychol. (2021) 12. 10.3389/fpsyg.2021.637839 33732197 PMC7957048

[33] Simonton KL Garn AC. Negative emotions as predictors of behavioral outcomes in middle school physical education. Eur Phys Educ Rev. (2020) 26:764–81. 10.1177/1356336X19879950

[34] Baños RM Escobar P Cebolla A Guixeres J Alvarez J Francisco J. Using virtual reality to distract overweight children from bodily sensations during exercise. Cyberpsychol Behav Soc Netw. (2016) 19:115–9. 10.1089/cyber.2015.0283 26882326

[35] Mestre DR Maïano C Dagonneau V Mercier CS. Does virtual reality enhance exercise performance, enjoyment and dissociation? An exploratory study on a stationary bike apparatus. Presence Teleoper Virtual Environ. (2011) 20:1–14. 10.1162/pres_a_00031

[36] Wininger SR Gieske DE. Measures of attentional focus: cognitive interviews and a field study. Athletic Insight. (2010) 2:125–46.

[37] Witmer BG Singer MJ. Measuring presence in virtual environments: a presence questionnaire. Presence. (1998) 7:225–40.

[38] Daly J Sindone AP Thompson DR Hancock K Chang E Davidson P. Barriers to participation in and adherence to cardiac rehabilitation programs: a critical literature review. Prog Cardiovasc Nurs. (2002) 17:8–17. 10.1111/j.0889-7204.2002.00614.x 11872976

[39] da Silva TD de Oliveira PM Dionizio JB de Santana AP Bahadori S Dias ED. Comparison between conventional intervention and non-immersive virtual reality in the rehabilitation of individuals in an inpatient unit for the treatment of COVID-19: a study protocol for a randomized controlled crossover trial. Front Psychol. (2021) 12:622618. 10.3389/fpsyg.2021.622618 33716889 PMC7943618

[40] Garcia-Bravo S Cano-de-la-Cuerda R Dominguez-Panigua J Compuzano-Ruiz R Barrenada-Copete E Lopez-Navas MJ. Effects of virtual reality on cardiac rehabilitation programs for ischemic heart disease: a randomized pilot clinical trial. Int J Environ Res Public Health. (2020) 17, 8472. 10.3390/ijerph17228472 33207670 PMC7696478

[41] Chen YL Chen CC Hsia PY Lin SK. Relationships of borg's RPE 6-20 scale and heart rate in dynamic and static exercises among a sample of young taiwanese men. Percep Motor Skills. (2013) 117:971–82. 10.2466/03.08.PMS.117x32z6 24665812

[42] Glass SC Knowlton RG Becque MD. Accuracy of RPE from graded exercise to establish exercise training intensity. Med Sci Sports Exerc. (1992) 24:1303–7. 10.1249/00005768-199211000-00017 1435183

[43] Lewis BA Williams DM Frayeh AL Marcus BH. Self-efficacy versus perceived enjoyment as predictors of physical activity behavior. Psychol Health. (2016) 31:456–69. 10.1080/08870446.2015.1111372 26541890 PMC4769927

[44] Michael SL Coffield E Lee SM Fulton JE. Variety, enjoyment, and physical activity participation among high school students. J Phys Act Health. (2016) 13:223–30. 10.1123/jpah.2014-0551 26107142 PMC5295133

[45] Farrow M Lutteroth C Rouse P Bilzon J. (2019). Virtual-reality exergaming improves performance during high-intensity interval training. Eur J Sport Sci. 19, 719–727. 10.1080/17461391.2018.1542459 30403927

[46] Garn AC Baker BL Beasley EK Solmon MA. What are the benefits of a commercial exergaming platform for college students? Examining physical activity, enjoyment, and future intentions. J Phys Activ Health. (2012) 9:311–18. 10.1123/jpah.9.2.311 22368230

[47] Decker ES Ekkekakis P. More efficient, perhaps, but at what price? Pleasure and enjoyment responses to high-intensity interval excise in low-active women with obesity. Psychol Sport Exerc. (2017) 28:1–10. 10.1016/j.psychsport.2016.09.005

[48] Marques M Alves E Henrique N Franchini E. Positive affective and enjoyment responses to four high-intensity interval exercise protocols. Percep Motor Skills. (2020) 127:742–65. 10.1177/0031512520918748 32323607

[49] Thin AG Poole N. Dance-based exergaming: User experience design implications for maximizing health benefits based on exercise intensity for perceived enjoyment. in Transactions of Edutainment IV. (2010). Berlin, Heidelberg: Springer. p. 189–99.

[50] Koslucher F Munafo J Stoffregen TA. Postural sway in men and women during nauseogenic motion of the illuminated environment. Exp Brain Res. (2016) 234:2709–20. 10.1007/s00221-016-4675-8 27236456

